# Total osteocalcin levels are independently associated with worse testicular function and a higher degree of hypothalamic–pituitary–gonadal axis activation in Klinefelter syndrome

**DOI:** 10.1007/s40618-024-02390-7

**Published:** 2024-05-21

**Authors:** F. Carlomagno, V. Hasenmajer, M. Spaziani, M. Tenuta, F. Sesti, C. Tarantino, C. Pozza, A. M. Isidori, D. Gianfrilli

**Affiliations:** 1 Section of Medical Pathophysiology, Food Science and Endocrinology, Department of Experimental Medicine, Sapienza University of Rome, 00161 Rome, Italy; 2 Endocrine and Andrological Regional Rare Disease Center (Endo-ERN Accredited), Policlinico Umberto I, 00161 Rome, Italy

**Keywords:** Osteocalcin, Klinefelter syndrome, Bone-testicular axis, Testosterone, Gonadotropins, Bone, Testis

## Abstract

**Purpose:**

The role of osteocalcin (OCN) in pubertal development, male hypogonadism, and the effect of testosterone (Te) replacement therapy (TRT) remains unclear. We aimed to investigate the total OCN (tOCN) concentrations in male patients with Klinefelter syndrome (KS), a model of adult hypergonadotropic hypogonadism.

**Methods:**

This retrospective longitudinal study investigated 254 male patients with KS (47,XXY) between 2007 and 2021 at an academic referral center, categorized as (1) prepubertal, (2) pubertal, and (3) adults. All prepubertal patients were Te-naïve. Adult patients were subcategorized as (1) eugonadal, (2) hypogonadal, and (3) receiving TRT. We also analyzed 18 adult patients with available tOCN levels before and 3 months after TRT commencement.

**Results:**

The tOCN levels varied throughout the lifespan according to pubertal status, were highest in eugonadal and significantly lower in TRT subjects, correlated with both LH (*p* = 0.017) and FSH levels (*p* = 0.004) in adults, and significantly declined after 3 months of TRT (*p* = 0.006) in the adult KS cohort. HPG-axis hormones levels demonstrated no correlation in prepubertal boys. Adjustment for age and body mass index confirmed previous results and revealed significant inverse correlations with total Te (*p* = 0.004), calculated free Te (*p* = 0.016), the Te/LH (*p* = 0.010), and calculated free Te/LH ratios (*p* = 0.031).

**Conclusion:**

In KS, a model of male hypergonadotropic hypogonadism, tOCN levels were not associated with gonadal function during normal prepuberty and pubertal development but were associated with worse testicular function and a higher degree of HPG stimulation in adults. TRT acutely reduced tOCN levels in adults.

## Introduction

The role of bone tissue as a versatile endocrine organ has clearly emerged recently. Osteocalcin (OCN), an osteoblast-derived polypeptide [1, 2], regulates multiple endocrine and physiological functions, including glucose and electrolyte homeostasis, muscle function, cardiovascular tone, cognitive abilities, and acute stress response [1, 3–8].

Specifically, OCN acts on peripheral tissues, such as pancreatic β cells and muscular and adipose tissues, by binding to its G-protein-coupled receptor 6a (Gpcr6a) to influence insulin secretion and peripheral metabolism. More recently, the role of OCN has appeared in terms of testicular function, and its action on the hypothalamic–pituitary–gonadal (HPG) axis, specifically on Leydig cells, stimulates testosterone (Te) production and peripheral release in the context of a *bone-testicular axis* [3, 9–11] in both adult male mice and humans [3, 9]. Specifically, OCN promotes testicular cell proliferation during development and testicular steroidogenesis in adult mice [12]. Furthermore, besides its direct regulation, it also promotes the expression of the receptor for luteinizing hormone (LH), LHCGR, thereby modulating LH signaling in Leydig cells [12]. Notably, OCN crosses the blood–brain barrier and binds to the Gpr158 receptor in the central nervous system, with accumulating evidence of its role in brain development, cognitive function, and motor coordination [13–16].

OCN contains three gamma-carboxyglutamic acid (Gla) residues, which undergo carboxylation by γ-glutamyl carboxylase in a vitamin K-dependent process, thereby conferring greater tertiary stability and affinity for Ca^2+^ and hydroxyapatite crystals [17]. Hence, OCN is well recognized as a constitutive bone matrix protein and a serum marker for bone formation and remodeling [16, 18, 19]. OCN reaches the bloodstream in its carboxylated form (cOCN) as well as in an uncarboxylated form (uOCN). The latter shows a lower affinity for the mineral matrix after undergoing decarboxylation. The total OCN (tOCN) pool thus consists of cOCN and uOCN.

In vitro and in vivo preclinical studies conducted by the Karsenty group first reported evidence for the role of OCN on the male gonad development and function. They demonstrated how the supernatant of osteoblast cultures significantly augmented Te production by Leydig cells and testis explants from mice. They later revealed how OCN^−/−^ male mice (both global and osteoblast-specific) bred poorly, demonstrating lower circulating Te levels, decreased testis size, reduced spermatogenesis, and increased LH levels. An opposite phenotype was observed in an OCN gain-of-function model, the Esp^−/−^ mouse [9]. In vitro studies have also described an increase in cOCN and uOCN release in culture medium by human adipose tissue upon stimulation with dihydrotestosterone (DHT), an effect which is blunted when cells are co-incubated with flutamide [20]*.*

However, studies on human subjects have reported conflicting results. Some cross-sectional and longitudinal studies have confirmed a relationship between OCN and Te. In particular, Liao et al. revealed that both total and free Te correlated with tOCN in 2400 adult males [21]. Similarly, Kirmani et al. detected a direct linear correlation in mid-pubertal boys [22]. However, population studies have yielded inconsistent results. In a German study of 1338 male individuals, tOCN showed a positively small, although significant, correlation with Te levels [23]. A second population study that was part of the Longitudinal Aging Study Amsterdam on 614 males aged 65–88 years revealed a positive correlation, limited to the highest quartile, for serum OCN with LH but not with Te [24]. Lastly, a third population study of 2966 older males aged 70–89 years reported no association between OCN and Te and an inverse relationship between uOCN and estradiol [25].

Few studies have so far investigated the *bone-testicular axis* in the context of male hypogonadism. Specifically, a Chinese study assessed subjects affected by idiopathic hypogonadotropic hypogonadism, observing mild positive associations between tOCN and maximum Te levels after hCG stimulation testing, alongside with a negative association with testicular volume [26]. A different Italian study focused on exploring the role of OCN in men affected by spinal cord injury, exhibiting a high prevalence of mostly hypogonadotropic hypogonadism. The authors observed a positive correlation between both total and cfT and tOCN concentrations, which persisted after adjusting for potential confounders [27]. In a prospective longitudinal study, obese subjects undergoing bariatric surgery, mostly hypogonadal, showed an increase in Te, tOCN and uOCN concentrations after 9 months, and the variation in tOCN concentrations was the only significant independent predictor of cfT recovery in hypogonadal men, after adjustment for age and BMI [28]. Lastly, in a Danish study the authors assessed serum INSL3 levels, reflecting Leydig cell function, in 70 men with Klinefelter syndrome (KS), to explore associations with bone metabolism markers, body composition, glucose and lipid metabolism. Of interest, INSL3 concentrations were significantly reduced in the whole KS cohort, and especially in the TRT-treated subgroup, compared to control men, and were positively associated with tOCN in untreated KS men only [29].

These incomplete and partly conflicting results represent the rationale of the present study, which aims to assess the relationship of tOCN with the HPG axis and testicular endocrine function in a large cohort of subjects with KS, as a model of hypergonadotropic hypogonadism during the life course. Taking advantage of a longitudinal approach, we retrospectively reviewed clinical and hormonal data to identify the relationship between Te and OCN in male hypogonadism.

## Patients and methods

### Study participants

This study was conducted at an academic referral center for patients with KS. The inclusion criteria were (1) patients with a confirmed diagnosis of classic, nonmosaic KS (47,XXY) based on peripheral blood karyotype analysis; (2) availability of gonadal function test results (LH, FSH, total Te, and SHBG) and tOCN levels, and (3) availability of concurrent clinical data. The exclusion criteria were (1) the presence of other known genetic conditions or chromosomal abnormalities; (2) the use of drugs, other than Te, that are active on the HPG axis or that may interfere with gonadal function tests; and (3) a history of surgery or radiotherapy on the testes or pituitary gland. Initially, we screened 274 patients with KS, of whom 20 infants, aged < 1 year, were excluded to prevent any potential influence of mini puberty on gonadal function. Thus, this study enrolled 254 patients with KS who were categorized according to Tanner stage and age into (1) prepubertal (n = 48, from 1 year of age until Tanner stage II), (2) pubertal (n = 46, Tanner stages II through V, < 18 years), and (3) adult (n = 160, Tanner stage V, ≥ 18 years) groups. Adult patients with KS were further subcategorized as (1) eugonadal (Te > 10.4 nmol/L; n = 47), (2) hypogonadal (Te < 10.4 nmol/L; n = 39), and (3) those receiving Te replacement therapy (TRT, n = 74). TRT was administered either transdermally or through various Te injections. Among the subgroup of adult subjects undergoing TRT (n = 74), we compared tOCN levels in subjects before and after initiating therapy, at variable time points, at least 6 months apart. Furthermore, we compared the ‘acute’ change in tOCN values in 18 men who underwent testing both before and 3 months after TRT initiation with Te undecanoate of 1.000 mg intramuscularly (i.m.) every 12 weeks (with a boosting dose after 6 weeks). A complete description of the present cohort has been published previously [30].

### Hormonal evaluation

Blood samples were obtained in the early morning (07:30–09:30 h) after an overnight fast, immediately centrifuged, and frozen at − 20 ℃. LH, FSH, Te, and SHBG were measured in duplicate using a chemiluminescent microparticle immunoassay (CMIA, Architect System, Abbott Laboratories, IL, USA) with limits of detection (LODs) of 0.07 mIU/mL, 0.05 mIU/mL, 0.1 nmol/L, and 0.28 nmol/L, respectively. The intra- and inter-assay coefficients of variation (CV) were 3.8% and 5.5% at 4.1 mIU/mL of LH, 3.6% and 5.4% at 3.2 mIU/mL of FSH, 5.65% and 2.1% at 10.08 nmol/L of Te, and 9.54% and 3.6% at 8.8 nmol/L of SHBG. Further, tOCN values were measured using a radioimmunometric assay (RIA, OSTEO-RIACT, Cisbio Bioassays, France) with LODs of 0.4 ng/mL and intra- and inter-assay CVs of 2.8% and 4.0%, respectively, at 29.6 ng/mL. We used Vermeulen’s formula to calculate the free Te concentrations (cfTe) from Te and SHBG levels [31]. We derived Te/LH and the cfTe/LH ratios as markers of Leydig cell function [32].

### Statistical analysis

Data are expressed as means and/or medians, as appropriate, and as standard deviations, 95% confidence intervals (CIs), and 25–75% interquartile ranges (IQR). Body surface area was calculated according to Haycock’s formula for all age groups [33]. The data distribution was visually inspected by analyzing histograms and normality plots. Brown-Forsythe and Welch analysis of variance tests were used to evaluate data for unequal variances (with regards to tOCN values among subjects grouped per pubertal and gonadal status), corrected for multiple comparisons (Dunnett T3), and with partial correlations after bootstrapping of 2000 samples. Paired comparisons were conducted using the Wilcoxon matched-pairs signed-rank test (with regards to tOCN values among subjects pre-vs. post-TRT commencement). We report Pearson’s correlation coefficient (*r*) with bootstrapping on 2000 samples in the text, and the simple linear regression best-fit lines, alongside their 95% confidence bands in Fig. 2. The statistical significance level was set at 0.05 and adjusted p-values are reported in the manuscript and Figures. Data are visually represented with box–whisker plots as the median (black lines), 25–75% IQR (boxes), and 2.5–97.5th percentiles (whiskers), with before–after graphs and scatter plots representing the best-fit line and its 95% CI for significant linear regressions. All statistical computations were conducted using IBM Statistical Package for the Social Sciences Statistics for Windows (version 28.0.1.1, IBM Corp.) and GraphPad Prism for Windows (version 8.3.0, GraphPad Software, LLC).

Written informed consent (or assent for underage minors) was obtained from all the patients (and/or parents), and controls. The study protocol conformed to the ethical guidelines of the Declaration of Helsinki, as approved by the Sapienza University Ethics Committee (ref. no. 6478, protocol no. 1038/21).

## Results

Table 1 shows the age, anthropometric parameters, gonadal function tests, and tOCN values in subjects with KS according to pubertal status, as extensively discussed in our previous paper [30].

**Table 1:** General characteristics, gonadal status, and total OCN levels in subjects with KS

	Prepubertal KS	Pubertal KS	Adult KS
**N**	48	46	160
Age, year	5.63 ± 3.86	13.57 ± 2.05	36.86 ± 11.27
Height, m#	1.16 ± 0.28	1.64 ± 0.21	1.80 ± 0.08
Weight, kg	24.02 ± 11.20	56.63 ± 15.00	86.45 ± 20.01
Body mass index, kg/m^2^	16.60 ± 2.55	19.10 ± 3.11	26.50 ± 5.35
Body surface area, m^2^	0.87 ± 0.31	1.50 ± 0.36	2.08 ± 0.27
**Gonadal function**			
LH, mUI/mL	0.72 ± 3.13	6.48 ± 5.39	13.46 ± 9.19
FSH, mUI/mL	1.58 ± 6.11	16.68 ± 13.43	23.07 ± 14.68
Te, nmol/L	1.05 ± 3.04	13.95 ± 8.67	14.20 ± 8.17
SHBG, nmol/Lo	116.80 ± 38.10	52.54 ± 24.76	42.00 ± 21.73
cfTe, pmol/L	10.96 ± 46.24	260.5 ± 266.6	268.9 ± 173.9
Total OCN, pg/mL	85.90 ± 30.40	130.00 ± 77.20	22.90 ± 9.00

Data are presented as means ± standard deviations. # length is reported for 7 non-standing children in place of height. *cfTe* calculated free testosterone; *KS* Klinefelter syndrome; *OCN* osteocalcin

### Total osteocalcin levels according to pubertal status

**Fig. 1:**
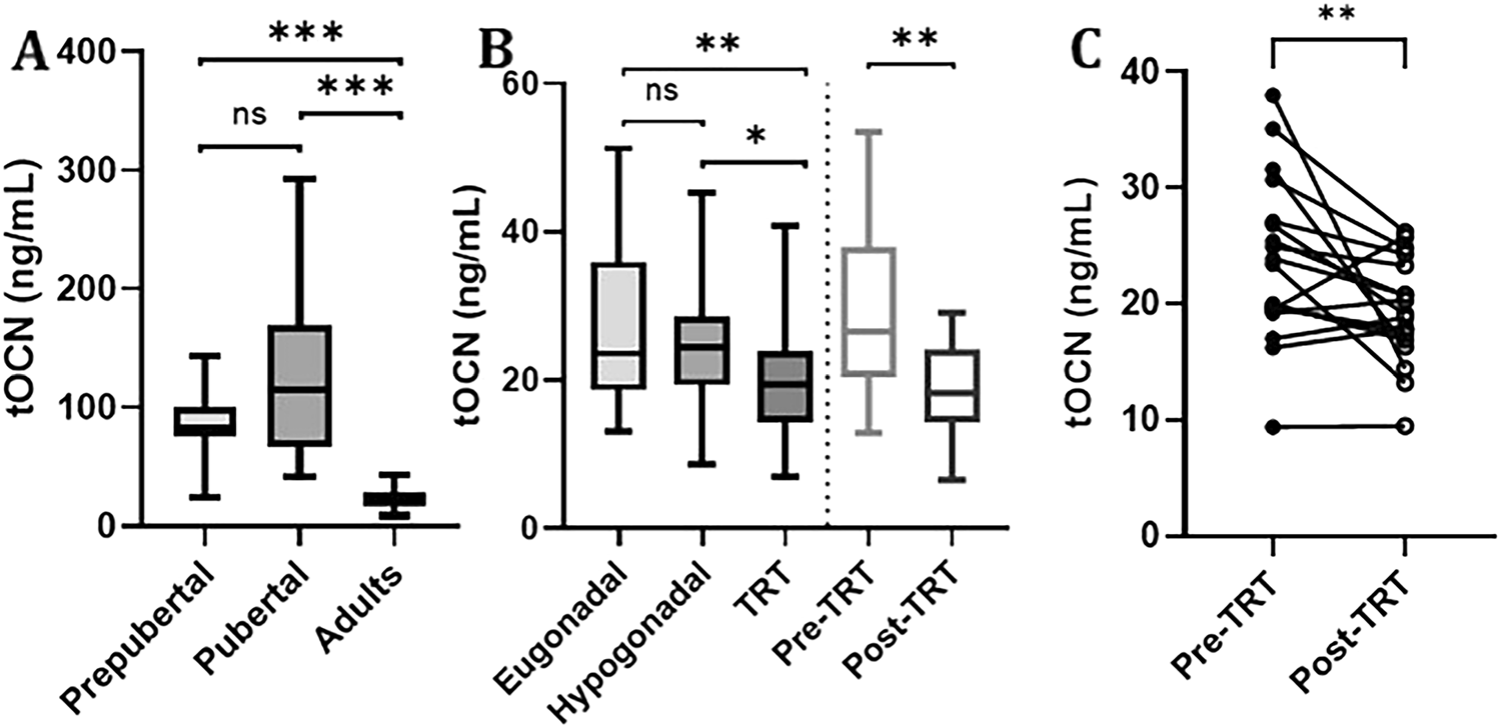
Total osteocalcin levels according to pubertal, gonadal, and TRT status in subjects with KS. Data are visually represented with box–whisker plots as the median (black lines), 25–75% IQR (boxes), and 2.5th–97.5th percentiles (whiskers), and with a before–after plot (panel **C**). tOCN values in subjects with KS are presented according to pubertal (panel **A**), gonadal (panel **B**), and TRT status (panels **B** and **C**). **A** number of subjects for each subgroup, according to pubertal status: prepubertal (n = 48), pubertal (n = 46), adult (n = 160). **B** number of adult subjects, according to gonadal status: eugonadal (n = 47), hypogonadal (n = 39), those receiving TRT (n = 74). **C** number of subjects in the pre- / post-TRT subgroups (n = 18). *p < 0.05, **p < 0.01, ***p < 0.001, ns = not significant. *KS* Klinefelter syndrome, *tOCN* total osteocalcin, *TRT* testosterone replacement therapy

As expected, tOCN levels varied throughout the lifespan of subjects with KS (Fig. 1A**)**. Prepubertal infants presented mean tOCN levels of 85.9 ± 30.4 ng/mL, peaking at 130.0 ± 77.2 ng/mL in pubertal children (*p* = 0.243 vs. prepubertal), then declining to 22.9 ± 9.0 ng/mL in adults (*p* < 0.001 vs. prepubertal and pubertal).

### Total osteocalcin levels according to gonadal status

Figure 1B shows the differences in tOCN values among adult subjects with KS. Values were highest in patients with eugonadism (26.5 ± 10.4 ng/mL), slightly lower in hypogonadism (24.5 ± 8.1 ng/mL, *p* = 0.268 vs. eugonadism), and significantly lower in subjects undergoing TRT (20.4 ± 8.0 ng/mL, *p* = 0.008 vs. eugonadism and *p* = 0.013 vs. hypogonadism).

The comparison of the change in tOCN values among adult subjects with KS with available pre- (28.5 ± 10.8) and post-TRT (21.7 ± 8.5) measurements, at least 6 months apart, revealed significantly lower tOCN levels at a mean 6.7 ± 5.1 years post-TRT commencement (*p* = 0.001) (Fig. 1B). Furthermore, a comparison of the smaller cohort of subjects with available paired measurements, before and 3 months after TRT commencement with 1000 mg of Te undecanoate i.m. (n = 18) revealed significantly reduced tOCN values (*p* = 0.006) from a median of 23.7 (IQR 19.4–28.0) to 19.0 (16.8–23.6) (Fig. 1C**)**.

### Relationship between total osteocalcin levels and the HPG axis

**Fig. 2:**
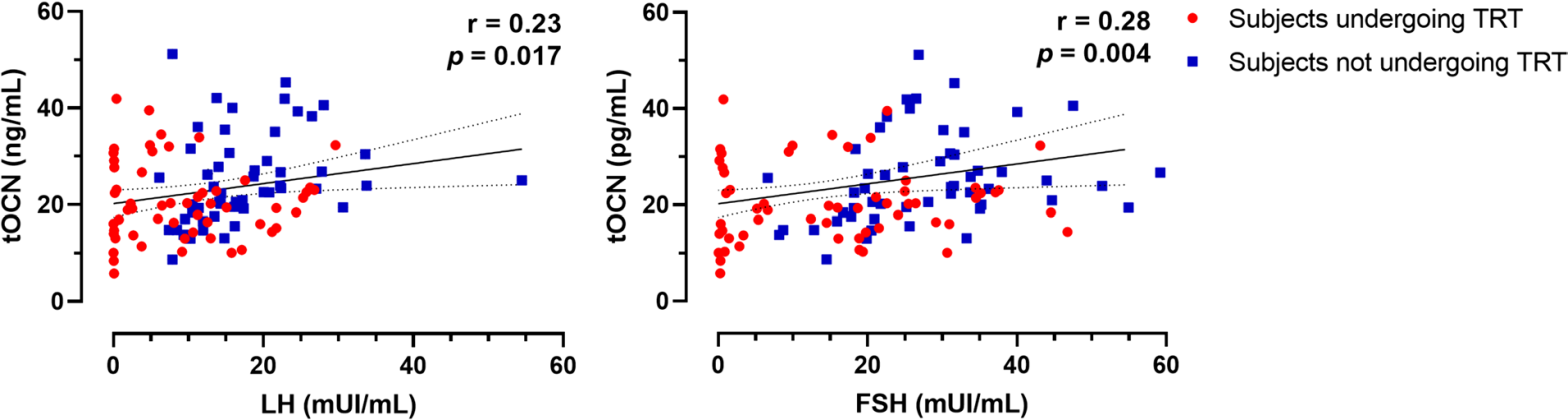
Total osteocalcin correlations with gonadotropins’ levels in adult subjects with KS Pearson’s coefficients and the respective *p*-values are shown for LH (panel **A**) and FSH (panel **B**). Subjects undergoing TRT are shown as red circles, whereas subjects not undergoing TRT are shown as blue boxes.** A **and** B**, number of subjects included in the analyses, n = 160. *tOCN* total osteocalcin

We also assessed the relationship between tOCN values among the different pubertal stages and the HPG-axis hormones and derived indices (LH, FSH, Te, SHBG, cfTe, Te/LH ratio, and cfTe/LH ratio). The groups of prepubertal and pubertal boys demonstrated no significant association between tOCN levels and HPG-axis hormones. Exploring the relationship between HPG-axis hormones and tOCN levels in the whole adult KS cohort, both LH (*r* = 0.23, *p* = 0.017) and FSH levels (*r* = 0.28, *p* = 0.004) demonstrated significant positive correlations with tOCN (Fig. 2A, B). Notably, these significant associations were maintained after the exclusion of subjects on TRT, both with LH (*r* = 0.21, *p* = 0.019) and FSH levels (*r* = 0.15, *p* = 0.014).

Surprisingly, when adjusting for age and body mass index (BMI), which significantly differed among groups according to gonadal status [30] previous results were confirmed and further revealed significant inverse correlations between tOCN and Te (*r* = − 0.44, *p* = 0.004), cfTe (*r* = − 0.37, *p* = 0.016), Te/LH ratio (*r* = − 0.40, *p* = 0.010), and cfTe/LH ratio (*r* = − 0.33, *p* = 0.031).

## Discussion

During the last decade, the role of bone as an endocrine organ has emerged and caused the conceptualization of a novel hormonal *bone-testicular axis* [34]. This axis moves from the established knowledge of the positive effects of androgens on both bone mineralization and resorption processes, thereby contributing to the achievement of peak bone mass and determining bone mineral density during the lifespan. Novel evidence has appeared concerning a feedback loop from the bone to the HPG axis through OCN, which is a small polypeptide (49 amino acids) well known as a noncollagenous bone matrix constituent. OCN is produced by osteoblasts and released into circulation (both directly and through active bone resorption) and has been convincingly shown in original experiments on both loss-of-function and gain-of-function OCN mouse models to be involved in peripheral tissue physiology, including muscle, adipose tissue, and pancreas, among others, by binding to the Gpcr6a receptor. Gpcr6a has been demonstrated in Leydig cells in the testis, and its stimulation by OCN influences testosterone production, testicular development, spermatogenesis, and breeding [3, 9]. Two separate research groups have recently investigated these results, which independently developed OCN-deficient mice and did not recapitulate the original endocrine phenotype [35, 36]. Subsequent debates focused on the possible influence of different genetic and environmental backgrounds [37–39]. However, in a recent paper by the Karsenty group, the authors were able to replicate metabolic, steroidogenic, and brain effects in mice of various genotypes and origins, in collaboration with an independent Chinese research laboratory, maintained on different genetic backgrounds, expanding on the differential role of embryonic vs. maternal OCN origin in developing and maintaining long-term organismal homeostasis [12].

Little is known about the human physiology and male hypogonadism. We decided to study subjects affected by KS as a model of hypergonadotropic hypogonadism and, as such, exhaustion of the LH-Te feedback loop. Bone metabolism is often affected in subjects with KS, as expected in a condition characterized by frequent overt hypogonadism. Specifically, fractures and reduced bone mass (osteoporosis) are frequently reported in KS [40–42], and bone mineral density (BMD) is frequently reduced. Bone quality assessments conducted using novel techniques, such as peripheral quantitative computed tomography (pQCT), have demonstrated low volumetric BMD in KS and reduced trabecular density [43]. Conversely, bone turnover markers, comprising both bone formation and resorption markers, are comparable to controls [44]. However, the role of TRT in preventing or reverting these changes in KS remains unclear. Hence, we aimed to evaluate the *bone-testicular axis*, focusing on the relationship between tOCN, hypothalamic–pituitary–gonadal (HPG) axis, and testicular endocrine function in the hypergonadotropic hypogonadal milieu of KS.

This large longitudinal cohort revealed increased tOCN levels from prepuberty into puberty, followed by a significant and marked decrease in adulthood. We then investigated the directionality of the supposed association between gonadal status and OCN, i.e., whether OCN promotes Te production by Leydig cells or if Te may be responsible for increased bone formation, thereby increasing serum tOCN levels. First, we observed that tOCN levels were lower in males with hypogonadism (hypotestosteronemic) compared to eugonadism (eutestosteronemic). This result can be explained by the known decline in tOCN concentrations with age and the significantly younger age of the eugonadal cohort compared to subjects with hypogonadism and undergoing TRT [30]. Moreover, not only was tOCN significantly reduced in subjects undergoing TRT compared to both the eugonadal and hypogonadal cohorts, but TRT significantly reduced circulating levels in our pre- and post-TRT analysis, thereby reinforcing the idea that increasing serum Te levels in males do not acutely increase serum tOCN levels.

We then studied the association between serum tOCN levels and HPG-axis hormones. We found no significant association in prepubertal children and pubertal boys with KS, which could be caused by the presence of an already evident, early HPG-axis impairment during mini puberty and prepuberty in this population [45]. Conversely, a small, although significant, positive association was present between tOCN and both LH and FSH levels in the entire adult cohort, which remained despite the exclusion of subjects undergoing TRT. Interestingly, adjusting for age and BMI, which significantly differed among groups according to gonadal status, confirmed the association between tOCN and gonadotropins and further revealed significant inverse correlations with Leydig cell function, specifically Te and cfTe concentrations, as well as with the Te/LH and cfTe/LH ratios. Hence, higher tOCN concentrations are associated with poorer testicular function and Leydig cell sensitivity and with a higher degree of HPG-axis stimulation in adults with KS.

Altogether, the available evidence supports the notion of the involvement of OCN in testicular function in males, both in conditions of physiological Te levels as well as during hypogonadal states, in a classic endocrine negative-feedback loop. Specifically, in the context of eugonadism, OCN purportedly acts by stimulating Te production both directly at the testis level (increasing Leydig cells steroidogenesis and LH sensitivity) [1, 9, 12, 46–50], as shown by large cohort and population studies [21–25], and indirectly at the central (hypothalamic-pituitary) level, inducing increased LH (and FSH) concentrations, as revealed in the present study. Conversely, Te acts by reducing OCN levels, as evidenced by the reduced circulating tOCN concentrations in subjects pre- and post-TRT (Fig. 1B, C). To the best of our knowledge, this is the first study describing the ‘acute’ effect of Te on tOCN concentrations in hypogonadic men. In fact, the available literature describing bone turnover markers in androgen deficient, non-diabetic men undergoing TRT had their closest time-point at 6 months after TRT start. Nonetheless, the available studies supported either an overall decrease in tOCN concentrations [51], or a differential trend based on baseline Te values above or below ~ 9.2 nmol/L [52], whereas concordant findings point to lower baseline tOCN values, which increase after TRT in type 2 diabetic men, who are however characterised by low bone turnover and insulin-resistance [51, 53].

It is plausible the positive effects of OCN on increasing Te levels are lost in the pathologic condition of HPG-axis ‘exhaustion’, such as the hypergonadotropic hypogonadism of KS, where higher levels of tOCN rather reflect a more severe state of hypogonadism, as shown by significant correlations with HPG-axis hormones after adjusting for confounding factors (Fig. 2A, B). The lack of negative feedback of (reduced) Te concentrations on tOCN production or release from the bone extracellular matrix may have caused this effect. These results are mostly in accordance with the scarce available literature investigating hypogonadic men. Specifically, in two studies investigating the *bone-testicular axis* in men affected by spinal cord injury (comprising subjects with ‘non-hypergonadotropic hypogonadism’) or idiopathic hypogonadotropic hypogonadism, a positive correlation was evidenced between tOCN and both basal total and cfT concentrations in the former group, which persisted after adjusting for potential confounders [27], whereas a positive correlation was evidenced with peak Te levels after hCG stimulation testing in the latter group [26]. Our results are also in line with a prospective study on obese subjects undergoing bariatric surgery, a condition characterised by functional hypogonadism, where the authors observed significant increases in (cf)Te, as well as both tOCN and uOCN concentrations after 9 months, with the increase in tOCN being the only independent predictor of hypogonadism recovery after multiple adjustments, further strengthening the interdependence between OCN and Te [28].

Lastly, the only available study so far to explore the bone-testicular axis in KS reported a positive association between tOCN values and serum INSL3 concentrations [29], a constitutive biomarker of Leydig cells differentiation status and number, being relatively insensitive to HPG axis control, or other acute factors [54]. Of interest, INSL3 concentrations were significantly reduced in the whole KS cohort, and especially in the TRT-treated subgroup, compared to control men, and were positively associated with tOCN in untreated men with KS only [29]. These results are not in contrast to the present findings, considering the authors did not assess an association between Leydig cells steroidogenic activity or sensitivity and tOCN concentrations, and in consideration of INSL3 being independent of the steroidogenic LH-mediated action [55, 56].

Our study presents some strengths as well as some limitations. This is the first study to thoroughly explore the *bone-testicular axis* in the context of hypergonadotropic hypogonadism across the lifespan, and it does so using KS as a clinical model. Second, our population was explored using a retrospective longitudinal approach at a single academic referral center for KS, and it evaluated subjects accounting for pubertal stage, gonadal status, and TRT administration. Conversely, we could not assess the specific role of uOCN, which has been proposed by some authors as the ‘metabolically active’ form of OCN, and we could not assess sex steroid levels using the current gold standard methodology of liquid chromatography–tandem mass spectrometry because of the retrospective nature of the study. Furthermore, the present study does not explore the bone health status of the enrolled KS subjects.

In conclusion, the present study is the first to explore the *bone-testicular-axis* in the context of hypergonadotropic hypogonadism in humans, using a large cohort of children, boys, and adult males with KS as a clinical model. We show how tOCN concentrations peak during pubertal development, demonstrate how Te exerts a negative effect on tOCN concentrations, reveal an inverse correlation between tOCN and Te output and a direct correlation between tOCN and gonadotropin levels, indicating worse testicular function, and propose a negative-feedback loop regulation of the *bone-testicular-axis*.

## Data Availability

Some or all datasets generated during and/or analyzed during the current study are not publicly available, but are available from the corresponding author on reasonable request.
